# Maternal autonomy but not social support is a predictor of child feeding indicators in the Northern Region, Ghana

**DOI:** 10.1186/s40795-022-00630-8

**Published:** 2022-11-18

**Authors:** Anthony Wemakor, Victoria Awuni, Salifu Issah

**Affiliations:** Department of Nutritional Sciences, School of Allied Health Sciences, University for Development Studies, P. O. Box TL 1883, Tamale, Ghana

**Keywords:** Maternal social support, Maternal autonomy, IYCF practices, Growth indicators, Tamale, Ghana

## Abstract

**Background:**

Child malnutrition may be mediated by poor infant and young child feeding (IYCF) practices. This study sought to explore if maternal social support or autonomy was related to IYCF indicators in Northern Region, Ghana.

**Methods:**

An analytical cross-sectional study was conducted with 395 randomly sampled mother–child pairs from 8 health facilities. Data were collected on socio-demographic characteristics, social support and autonomy statuses of mothers, and dietary intake and anthropometry of children. Maternal social support and autonomy statuses were derived and classified into tertiles and IYCF and child growth indicators were derived based on WHO protocol. Logistic regression analysis was used to explore the association of maternal social support and autonomy statuses to IYCF indicators and child nutritional status.

**Results:**

The mean age of the women was 27 (± 5.10) years and most belonged to the lowest tertile of social support (52.4%), and autonomy (44.1%). About half of the children, 53.2% and 44.6%, received Minimum Dietary Diversity (MDD) and Minimum Acceptable Diet (MAD) respectively but the majority (72.9%) received Minimum Meal Frequency (MMF). About a fifth of the children, 21.0%, 24.1%, and 20.5%, were wasted, stunted, and underweight respectively. Maternal autonomy was associated with IYCF but not growth indicators of young children. Compared to children of mothers of richest autonomy tertile, children of women of middle autonomy tertile were 67% less likely to receive MDD [Adjusted Odds Ratio (AOR): 0.33; 95% Confidence Interval (CI): 0.18–0.59], and 56% less likely to receive MAD (AOR: 0.44; 95% CI: 0.24–0.77). Also, children belonging to mothers of poorest autonomy tertile were 56% less likely to receive MMF compared to children of richest maternal autonomy tertile (AOR: 0.44; 95% CI: 0.23–0.84).

**Conclusion:**

Maternal autonomy and not social support is associated with IYCF indicators of children in Northern Ghana; child survival programmes should incorporate or strengthen women empowerment interventions to improve child nutrition.

**Supplementary Information:**

The online version contains supplementary material available at 10.1186/s40795-022-00630-8.

## Background

Despite the successes chalked in child malnutrition prevention, millions of children are still affected, and this continues to be a problem of public health concern. Globally, 144 million children under 5 are chronically undernourished, 40.0% in Africa [1], 19.0% in Ghana, and 33.1% in the Northern Region, which has the highest burden of child malnutrition in the country [2]. Childhood malnutrition accounts for 45% of the mortality rate in children below the age of five years [3]. Malnutrition can rob children of their potential and its consequences are not limited to health and survival, but also include long-term effects on intellectual growth, school performance, and ultimately future earning and productivity [4].

Reducing the burden of child malnutrition has been one of the major goals of the Ghana government and many international agencies in Ghana over the years. Identifying the risk factors of malnutrition would enable the Ghana Health Service and other stakeholders implement evidence-based policies and interventions to improve child nutrition and contribute to realizing this goal.

Inadequate dietary intake and infectious disease are the common immediate causes of undernutrition [5] but social factors including poverty, ignorance, large family size, fewer sleeping rooms, socio-economic conditions, and insufficient maternal care, support and autonomy have a role in its aetiology [6]. Social support to women helps to reduce psychosocial problems including depression and enhances health and survival in mothers and their children. It is the perception or experience that one is loved and cared for by others, esteemed and valued, and is part of a social network of mutual assistance and obligations [7]. It incorporates the beliefs and experiences of individuals about love and care extended to them by significant others such as partners, relatives, co-workers, friends, and social and community ties. According to a study in Zimbabwe, women with adequate social support are more likely to provide optimal care for their children [8] and maternal social support has been demonstrated to be associated with improved breastfeeding and complementary feeding [9]. Thus, the provision of social support to mothers could help improve child feeding practices and ultimately the nutritional status of young children.

Maternal autonomy is also a social factor that ultimately affects children’s health and growth. It is a concept that includes control of resources and information, decision-making on movement, and decision-making on one’s concerns and those of close relatives [10]. Autonomy is established when women can make decisions freely about what concerns them and their dependents and it makes access to food, land, income, and other sources of wealth and resources such as knowledge, power, and prestige within the family and community very easy. Globally, an innumerable number of women continues to enjoy very limited autonomy [11]. Autonomous women are more likely to use contraception, have prolonged birth spacing, and have less chance of indulging in unplanned pregnancies and therefore improved household food security [11]. On the other hand, a low level of women’s autonomy limits communication between partners about nutritional and other health-related matters thereby limiting women’s access to safe, healthy, and nutritious food leading to poor birth outcomes [12] and is associated with higher mortality, injuries, illness, and general health problems [13]. Some studies have reported associations between women’s autonomy and child feeding and/or nutritional status elsewhere [8, 12–15] and in Ghana [16]. A systematic review based on demographic and health survey data from 30 countries in Sub-Saharan Africa also found an association between maternal autonomy and child feeding and growth indicators [17].

Despite that maternal social support and autonomy have been found to influence the nutritional status of children, it is not clear how this influence is mediated for it to be harnessed in planning intervention programmes in Ghana. Also, inadequate evidence has not been reported on this association in Ghana, especially in the Northern Region which has the highest burden of child malnutrition in the country. Therefore, this study sought to examine whether maternal social support and autonomy were associated with infant and young child feeding (IYCF) practices, and secondarily with child growth indicators in Tamale Metropolis, Northern Region, Ghana. We hypothesized that child feeding mediates the association between maternal social support or autonomy and child growth.

## Methods

### Study design, site, and population

An analytical cross-sectional design was employed for the study in Tamale Metropolitan District, Ghana. The District has Tamale as its capital and has a population of 233,252 of which 50.3% are females and about 80.8% of the people dwell in the urban centers of the Metropolis [18]. Also, 60.1% of the residents aged 11 years or more in the metropolis are literates. The majority of inhabitants work in the service sector representing 33% while 8.1% of workers constitutes professionals. Islam and Dagomba are the predominant religion and ethnicity respectively in the Metropolis. Women and children aged 6–23 months who were attending Child Welfare Clinics (CWCs) in the metropolis were the target of the study.

### Sample size and sampling technique

Using the single population proportion formula [19], a sample size of 395 was estimated using the prevalence of childhood stunting (33.1%) in the Northern Region [2], 0.05 margin of error, and 95% confidence interval (i.e., 1.96). Eight health facilities (two each in four sub-metros): Tamale Reproductive and Child Health (RCH) clinic, Moshie Zongo clinic, Vitting health center, Zuo Community Health Planning and Services (CHPS) compound, Nyohani health center, Gbambaya CHPS compound, Bilpela health center and Banvim CHPS compound were randomly selected for the study. The estimated sample size (n = 395) was proportionally divided among the eight health facilities. The study participants were recruited and interviewed at the CWCs of the study health facilities using simple random sampling, which was applied using the lottery method. The lottery method was conducted by using pieces of paper with ‘yes’ or ‘no’ written on them and folded in a bowl and vigorously shaken. All mothers who consented to participate in the study were allowed to pick the folded pieces of paper one after the other. Any participant that selected ‘yes’ was recruited for the study until the required number per day per facility was exhausted.

### Data collection tool and procedures

A semi-structured questionnaire comprising of both open- and close-ended questions was used to gather information from participants. The tool consisted of researcher-constructed items and two standard scales [20, 21]. The questionnaire had sections on dietary intake and anthropometry of children and socio-demographic characteristics, social support and autonomy statuses of mothers. The interviews were conducted on-site by trained research assistants from January to February, 2021. The data collection is described below:

#### Dietary assessment

Data from 24-h dietary recall were used to construct three IYCF indicators: Minimum Dietary Diversity (MDD), Minimum Meal Frequency (MMF) and Minimum Acceptable Diet (MAD) based on WHO recommendations [22]. In the 24-h dietary recall, the mothers of the children were asked to recall the soft and semi-solid foods the children were fed and number of times of feeding in the last 24 h prior to the interview. Children who were fed from at least 4 of 7 designated food groups were coded as having received MDD. A child who was fed at least the recommended number of soft/semi-solid meals based on age and breastfeeding status in the last 24 h (Breastfeeding: 2 times for 6–8 months, 3 times for 9–23 months; Non-breastfeeding: 4 times for 6–23 months) was classifed to have received MMF. Children who received both MDD and MMF were coded as having received MAD. The MDD, MMF and MAD were the primary dependent variables of the study.

#### Anthropometry

Recumbent length was measured using an infantometre to the nearest 1 cm, weight was measured using an electronic scale, and age was deduced from date of birth recorded from CWC cards and date of survey. WHO anthtro was used to convert the anthropometric measurements into length-for-weight z-score, length-for-age z-score and weight-for-age z-score which were recoded into wasting, stunting and underweight respectively using a cut-off of less than -2 standard deviation units from the WHO multi-centre growth standard [23]. Stunting and wasting were the secondary dependent variables of the study.

#### Socio-demographic characteristics

Data were collected on socio-demographic characteristics of respondents and their children. Data on age of the women were presented in 3 age categories 15–24 years, 25–29 years and 30 years and above. The marital status of the women were used to create two categories “in a union” i.e., currently married and “not in a union” i.e., single and never married, divorced or widowed. Educational status was presented as no education, low education (i.e., primary school, vocational school, technical school, junior high school and senior high school) and high education which comprises tertiary level education. Ethnicity had two categories: Dagomba (the predominant ethnic group of the Northern Region) and all others; and religion also two categories, Islam (the predominant religion in the region) and all others. Information was also captured on type of building and the nature of residential area of the respondents. Using ownership of 14 household items, principal component analysis was used to derive household wealth index which was classified into three categories: poorest, middle and richest tertiles [24].

#### Assessment of maternal autonomy

Maternal autonomy was measured using the women’s involvement in decision-making in the household [20]. Five (5) questions on whether the women have a say in decision making concerning buying of household items and stationery for children, the number of children the couple should have and how the family’s income is to be spent were asked. A response attracted 2 points when a decision was made by a woman alone, 1 point when made by both husband and woman and no points when made by others. The possible maximum score on decision making autonomy was 10. The scores were arranged in ascending order and divided into 3 equal parts (tertiles). The women having scores in the highest tertile were classified as belonging to the richest tertile of autonomy, followed by those belonging to middle tertile of autonomy and poorest tertile of autonomy. This was one of the independent variables of the study.

#### Assessment of maternal social support

Maternal social support scale made up of six questions of five Likert scales [21] was used to measure social support of the women. The items are “I have good friends who support me” (1), “My family is always there for me” (2), “My husband or partner helps me a lot” (3), “There is a conflict with my husband or partner” (4), “I feel controlled by my husband or partner” (5), and “I feel loved by my husband or partner” (6). The responses for statements 1 – 3 and 6 are “Always”, “Most of the time”, “Some of the time”, “Rarely” and “Never” which were scored 5, 4, 3, 2, and 1 respectively. The statements 4 and 5 were reverse scored. The maximum score for the 6 items was 30, the total scores were arranged in ascending order and divided into 3 equal parts (tertiles). The women having scores in the highest tertile were classified as having the richest social support, followed by those having medium social support and poorest social support. This was one of the independent variables of the study.

### Data analysis

The data were analysed with Stata version 15/SE (Stata Corp, Texas, USA). Chi-square/Fisher’s exact test and logistic regression analysis were used to evaluate the association of maternal social support and autonomy to IYCF and child growth indicators, and to identify other determinants of IYCF and child growth indicators. In the search for association, independent variables that were significant in bivariate analyses were considered for multivariate analyses and adjusted odds ratios and their 95% confidence intervals were reported. The fit of the multivariate logistic regression models was evaluated using Hosmer–Lemeshow goodness of fit test. Statistical significance was declared at *p* < 0.05 for all analyses.

### Ethics approval and consent to participate

The methodology of this research conformed to the ethical principles of the Helsinki Declaration. Ethical clearance was obtained from Committee on Human Research, Publication and Ethics, Kwame Nkrumah University of Science and Technology, Ghana (CHRPE/AP/043/21). Written informed consent was obtained from the subjects before they were interviewed and assent from under-aged mothers. The participants were also informed that participation in the study was voluntary and they were at liberty to stop the interview if they were uncomfortable. Also, it was made clear to them that the data they were providing would be kept on password-protected computer and only use for the purpose of this research project.

## Results

### Socio-demographic characteristics, social support and autonomy of respondents

Most of the children in the study were in the age group 6–11 months (65.3%), Table 1. The mean age of the mothers was 27 (± 5.0) years and most had low education (54.7%). Most of the mothers practised Islamic religion (94.1%) or belonged to Dagomba ethnic group (82.5%), and majority lived in block houses (78.5%). About a third (33.7%) belonged to the poorest household wealth index tertile and about half (50.6%) lived in rural or peri-urban areas. With respect to social support and maternal autonomy, 52.4% and 44.1% belonged to the lowest tertiles respectively.

**Table 1:** Socio-demographic characteristics of respondents

**Characteristic**	**Frequency**	**Percent (%)**
**Child age (months)**		
6–11	258	65.3
12–17	79	20.0
18–23	58	14.7
**Maternal age (years)**		
15–24	132	33.4
25–29	139	35.2
30 +	124	31.4
**Marital status**		
In a union	373	94.4
Not in a union	22	5.6
**Educational status**		
No education	134	33.9
Low education	216	54.7
High education	45	11.4
**Ethnicity**		
Dagomba	326	82.5
Others	69	17.5
**Religion**		
Christianity	34	8.6
Islam	361	91.4
**Household wealth index (tertile)**		
Poorest	133	33.7
Medium	132	33.4
Richest	130	32.9
**Type of building**		
Block	310	78.5
Others	85	21.5
**Residential area**		
Rural	26	6.6
Urban	195	49.4
Peri-urban	174	44.1
**Maternal autonomy (tertile)**		
Poorest	174	44.1
Medium	138	34.9
Richest	83	21.0
**Maternal social support (tertile)**		
Poorest	207	52.4
Medium	125	31.6
Richest	63	15.9

### Association between maternal social support or autonomy and child feeding indicators

Of the children, 53.2% [95% Confidence Interval (CI): 48.1 – 58.2] received MDD, 72.9% (95% CI: 68.2 – 77.2) received MMF, and 44.6% (95% CI: 40.0 – 50.0) received MAD, Table 2. While social support status was not related to any indicator of child feeding, maternal autonomy status was related to all the three. Compared to the children of mothers of the richest autonomy tertile, children belonging to mothers of middle autonomy tertile were 63% and 54% less likely to receive MDD [Odds Ratio (OR) 0.37; 95% CI: 0.21–0.65] and MAD (OR 0.46; 95% CI: 0.27–0.81) respectively, and those belonging to mothers of the poorest autonomy tertile were 57% less likely to receive MMF (OR 0.43; 95% CI 0.23–0.82), Table 3. Age of child was associated with all the three indicators of complementary feeding (MDD, *p* = 0.001; MMF, *p* = 0.030 and MAD, *p* < 0.001 respectively), Supplementary Table 1, so was adjusted for in multivariate analyses. In the multivariate analyses, maternal autonomy retained significance. Children belonging to women classified to middle autonomy tertile compared to those of mothers of richest autonomy tertile were 67% less likely to receive MDD [Adjusted Odds Ratio (AOR): 0.33; 95% Confidence Interval (CI): 0.18–0.59], Table 4. The Hosmer–Lemeshow goodness of fit test was insignificant (*p* = 0.1160) suggesting that the model fitted the data well. Also, children belonging to mothers with poorest autonomy tertile were 56% less likely to receive MMF compared to children of richest maternal autonomy tertile (AOR: 0.44; 95% CI: 0.23–0.84). An insignificant Hosmer–Lemeshow goodness of fit test was obtained (*p* = 0.9855). Also, children belonging to mothers with middle autonomy tertile were 56% less likely to receive MAD compared to children of richest maternal autonomy tertile (AOR: 0.44; 95% CI: 0.24–0.77), Table 5. An insignificant Hosmer–Lemeshow goodness of fit test was obtained (*p* = 0.3838).

**Table 2:** Magnitude of infant and young child feeding indicators and child undernutrition

**Characteristic**	**Frequency**	**Percent (%)**	**95% Confidence Interval**
**Infant and young child feeding indicators**			
Minimum dietary diversity	210	53.2	48.1 – 58.2
Minimum meal frequency	288	72.9	68.2 – 77.2
Minimum acceptable diet	176	44.6	40.0 – 50.0
**Child nutritional status**			
Wasting	83	21.0	19.9 – 28.6
Stunting	95	24.1	17.1 – 25.4
Underweight	81	20.5	16.6 – 24.8

**Table 3:** Relationship between mother’s social support or autonomy, and infant and young child feeding indicators

**Characteristic**		**Minimum dietary diversity**			**Minimum meal frequency**			**Minimum acceptable diet**		
	Total	**Yes (%)**	**OR (95% CI)**	***P*-value**	**Yes (%)**	**OR (95% CI)**	***P*-value**	**Yes (%)**	**OR (95% CI)**	***P*-value**
**Maternal social Support (tertile)**										
Poorest	207	74.4	1.49 (0.85–2.63)	0.164	56.0	1.35 (0.73–2.50)	0.338	48.3	1.74 (0.97–3.13)	0.063
Medium	125	72.8	1.27 (0.69–2.33)	0.440	52.0	1.24 (0.64–2.41)	0.516	43.2	1.42 (0.76–2.65)	0.276
Richest	63	68.3	1.00		46.0	1.00		34.9	1.00	
**Maternal autonomy (tertile)**										
Poorest	174	66.1	0.64 (0.37–1.11)	0.110	55.7	0.43 (0.23–0.82)	0.010	47.1	0.75 (0.45–1.27)	0.288
Medium	138	76.1	0.37 (0.21–0.65)	0.001	42.0	0.70 (0.35–1.39)	0.309	35.5	0.46 (0.27–0.81)	0.007
Richest	83	81.9	1.00		66.3	1.00		54.2	1.00

**Table 4:** Multivariate analysis of the predictors of indicators of infant and young child feeding

**Variable**	**Adjusted Odds Ratio**	**95% Confidence Interval**	***P*-value**
**Minimum dietary diversity**			
**Maternal autonomy (tertile)**			
Poorest	0.68	0.39–1.19	0.181
Middle	0.33	0.18–0.59	< 0.001
Richest	1.00		
**Age of child (months)**			
6–11	1.00		
12–17	2.04	1.20–3.47	0.008
18–23	4.67	2.37–9.20	< 0.001
**Minimum meal frequency**			
**Maternal autonomy (tertile)**			
Poorest	0.44	0.23–0.84	0.012
Middle	0.70	0.35–1.40	0.318
Richest	1.00		
**Age group of children (months)**			
6–11	1.00		
12–17	2.01	1.07–3.76	0.030
18–23	1.77	0.86–3.63	0.119
**Minimum acceptable diet**			
**Maternal autonomy (tertile)**			
Poorest	0.80	0.47–1.37	0.410
Middle	0.44	0.24–0.77	0.005
Richest	1.00		
**Age group of children (months)**			
6–11	1.00		
12–17	2.21	1.31–3.71	0.003
18–23	3.22	1.75–5.92	< 0.001

**Table 5:** Relationship between mother’s social support or autonomy, and child wasting and stunting

**Characteristic**		**Stunting**			**Wasting**		
	**Total**	**Yes (%)**	**OR (95% CI)**	***P*-value**	**Yes (%)**	**OR (95% CI)**	***P*-value**
**Maternal social support (tertile)**							
Poorest	207	22.2	0.84 (0.44–1.62)	0.600	20.8	0.92 (0.46–1.82)	0.805
Medium	125	26.4	1.05 (0.53–2.11)	0.882	20.8	0.92 (0.44–1.92)	0.822
Richest	63	25.4	1.00		22.2	1.00	
**Maternal autonomy (tertile)**							
Poorest	174	20.7	0.82 (0.44–1.53)	0.537	20.7	1.09 (0.57–2.11)	0.792
Medium	138	28.3	1.24 (0.66–2.32)	0.498	22.5	1.21 (0.62–2.39)	0.575
Richest	83	24.1	1.00		19.3	1.00	

### Association between maternal social support or autonomy and child growth indicators

Assessment of nutritional status revealed that, 24.1% (95% CI: 17.1 – 25.4), 21.0% (95% CI: 19.9 – 28.6) and 20.5% (95% CI: 16.6 – 24.8) of the children were stunted, wasted and underweight respectively, Table 2. Both maternal social support and autonomy states were not related to any indicator of child growth (Table 5) but low birth weight was related to wasting, and child age and household wealth index were related to stunting (Table 6). The risk of stunting was higher in older children and children belonging to low wealth index households in both bivariate and multivariate analyses. Children belonging to low wealth index households were almost twice as likely to be stunted compared to those belonging to high wealth index households (AOR: 1.98; 95% CI: 1.06–3.70), and those aged 18–23 months compared to those aged 6–11 months were 9 times more likely to be stunted (AOR: 9.04; 95% CI: 4.75–17.21), Table 7. The Hosmer–Lemeshow goodness of fit test of the model was insignificant (*p* = 0.6124).

**Table 6:** Other determinants of child wasting and stunting

**Variable**		**Wasting**		**Stunting**	
	**Total**	**Yes (%)**	***P*-value**	**Yes (%)**	***P*-value**
**Low birth weight**			0.026		0.171
Yes	88	29.5		29.5	
No	307	18.6		22.5	
**Age group of children (months)**			0.411		< 0.001
6–11	258	19.8		12.8	
12–17	79	20.3		35.4	
18–23	58	27.6		58.6	
**Sex of children**			0.563		0.111
Male	184	22.3		27.7	
Female	211	19.9		20.9	
**Maternal age (years)**			0.167		0.192
15–24	132	17.4		28.0	
25–29	139	19.4		25.2	
30 +	124	26.6		18.5	
**Marital status**			0.382		0.507
In a union	373	21.4		24.4	
Not in a union	22	13.6		18.2	
**Educational status**			0.130		0.577
No education	134	19.4		24.6	
Low education	216	24.1		25.0	
High education	45	11.1		17.8	
**Religion**			0.949		0.08
Christianity	34	20.6		11.8	
Islam	361	21.1		25.2	
**Ethnicity**			0.870		0.421
Dagomba	326	20.9		24.8	
Others	69	21.7		20.3	
**Type of building mothers dwelt in**			0.967		0.679
Block	310	21.0		24.5	
Others	85	21.2		22.4	
**Household wealth index (tertile)**			0.243		0.005
Poorest	133	24.1		33.8	
Medium	132	22.7		20.5	
Richest	130	16.2		17.7

**Table 7:** Multivariate analysis of the predictors of stunting in children

**Variable**	**Adjusted Odds Ratio**	**95% Confidence Interval**	***P*-value**
**Child stunting**			
**Age group of children (months)**			
6–11	1.00		
12–17	3.58	1.98–6.50	< 0.001
18–23	9.04	4.75–17.21	< 0.001
**Household wealth index (tertile)**			
Poorest	1.98	1.06–3.70	0.032
Middle	1.13	0.58–2.19	0.727
Richest	1.00		

## Discussion

Some studies have reported associations between indices of maternal social support or autonomy and child growth indicators, but the mechanisms of these associations are unknown. In this study we investigated child feeding practices as a potential pathway linking maternal social support or autonomy and child growth indicators.

Less than half of the children received MAD, an indicator of adequate feeding frequency and dietary diversity, suggesting that parents are not feeding their children according to global recommendations. According to the latest Ghana Demographic and Health Survey, only 14.1% of children aged 6–23 months was fed MAD nationally [2] but the Multiple Indicator Cluster Survey Six conducted later estimated an even lower rate of MAD receipt (12.0%) [25]. The poor feeding practices of mothers may partly explain the high undernutrition rates in the country and in most developing countries and may constitute a mechanism by which maternal factors impact child growth and development.

While the rate of underweight in the study population (6–23 months old children) in the Metropolis was similar to what pertains in the Northern Region (6–59 months old children) (20.5% versus 20.0%), stunting was less prevalent within the Metropolis compared to the region (24.1% versus 33.1%) but wasting level was far higher in the Metropolis compared to the region (21.0% versus 6.3%) [2]. Both wasting and underweight rates within the Metropolis are within cut-offs stipulated by WHO to be declared as problems of public health significance i.e., 10–14% and 20–29% respectively [26]. Ghana Health Service and the non-governmental organisations intervening in the region must intensify their activities to salvage the situation. Since most interventions are food-based, the high prevalence of child undernutrition after several years of intervening may be a wake-up call to investigate new risk factors and include them in programmes if any meaningful progress is to be made in the fight against malnutrition.

A little less than half of the women studied were classified into the poorest tertile of social support. The proportion of mothers who receive adequate social support varies widely and was reported to be 52.5% in Ethiopia [27], and 58% in Kenya [28]. Across the literature, many constructs and scales were used to measure social support making comparability of the reported prevalence rates very difficult. Social support plays a buffering role from stressful life events in pregnancy [29] and is associated with positive child feeding behaviours in the postpartum period [8, 9]. We hypothesized that adequate social support would translate into household food security, healthy environment and access to health services and ensure that children attain their growth potential. The level of social support given to women by significant others in the study area must be increased in order to improve the women’s ability to adequately play their nurturing roles and provide a healthy and stimulating environment for adequate growth.

Most of the women studied belonged to the poorest tertile of autonomy but a similar study like ours found a slightly higher prevalence of overall autonomy status (52.8%) of women in Bawku West District of Upper East Region [16]. Our rate is however similar to rates reported in Bangladesh (42.4%) [14] and India (40%) [30] for maternal autonomy but the comparison is challenged because of the various ways maternal autonomy was defined and measured in the literature. The level of adequate maternal autonomy measured in the study population suggests that there is the need to bring to the fore and increase women empowerment campaigns and activities in the region. This may contribute to a reduction in the rate of undernutrition in the region.

Some previous studies have reported an association between maternal social support and child feeding indicators [8, 12–17] but others did not e.g., [12]. We did not find an association between maternal social support status and child feeding indicators and it is not clear what explains the lack of association between these constructs in the study area. However, it is possible that there is no association between these constructs in the population studied.

Children belonging to mothers with more autonomy were more likely to have been fed adequately in terms of dietary diversity and meal frequency suggesting poorly autonomous mothers were more likely to engage in improper feeding practices and hence predispose their children to malnutrition. We hypothesized that highly autonomous women would be able to feed their children according to global recommendations and found evidence to support this in our study. Elsewhere in Ghana, financial decision-making autonomy was associated with MAD receipt by children [16] but this association is not supported by all authors e.g., [31]. The discrepancy in the link between maternal autonomy indices and measures of dietary practices in children may be explained by the variety of constructs and scales used in measuring maternal autonomy and child feeding indicators. However, it is possible that feeding practices mediate maternal autonomy indices and child growth indicators.

In addition to the association between maternal autonomy and child feeding indicators, we found that child age was associated with all the three indicators of complementary feeding (MDD, MMF and MAD) with older children being more likely to receive them compared to younger ones in the 24 h preceding the survey. Complementary feeding is an important developmental milestone in young children occurring at a formative time for the development of healthy eating habits [32]. Late introduction of complementary foods and the time it takes for infants to get used to it when introduced may explain the observation that older children are more likely to have received MDD, MMF and MAD.

In our study, neither maternal social support nor autonomy was associated to child wasting or stunting. Association between women’s social support or autonomy and child nutritional status has been conflicting over the years, there is an association between the constructs in some studies [33, 34] but not in others [16, 35]. Similarly, some research also found association between maternal autonomy and child nutritional status [8, 12–17] while some did not e.g., [31]. The diversity in the meaning and measurement of the constructs involved in these studies may partly explain these conflicting observations.

However, we found that low birth weight predicted wasting status, and child age and household wealth index predicted stunting status. Low birth weight is a known risk factor for child wasting [36, 37] and must be controlled for success to be achieved in wasting reduction. Stunting, a form of chronic malnutrition resulting from prolonged exposure to inadequate dietary intake and infections, is more prevalent in older children peaking around 24–35 months [38] and children of poor wealth index households [39]. Stunting is reflective of the hydra-headed nature of malnutrition and a variety of factors at individual, community and population levels must be tackled to reduce its incidence and prevalence.

The observation that maternal autonomy predicts child feeding indicators could have policy implications for the fight against childhood malnutrition as maternal autonomy may be related directly or indirectly to child feeding and growth. Firstly, further research is needed to determine if the observed association between maternal autonomy and child feeding indicators is a cause-and-effect relationship. Secondly, programmes that target or aim to improve child feeding practices should have a set of interventions targeted at promoting maternal autonomy in addition to other interventions targeting the immediate and basic determinants of child nutritional status.

### Strength and limitations

We used a team of experienced enumerators who have a minimum of bachelor’s degree in nutrition for the interviews and anthropometry ensuring that data were of very high quality. A cross-sectional study measuring both exposure and outcome at the same time was conducted meaning the findings cannot portray causation. There are no reports of the scales used to measure maternal social support and autonomy being validated in Ghana and their reliability in the Ghanaian context can only be extrapolated. There may be recall bias arising from forgetfulness and an attempt to please enumerators. Comparison of IYCF indicators used figures for children 6–59 months while our study population was children aged 6–23 months. Also, the generalizability of the study to other regions or the entire country may be limited due to differences in socio-demographic and maternal characteristics. Despite these limitations, the manuscript reports a novel study investigating the association of two maternal capability constructs to child feeding and growth indicators.

## Conclusion

Maternal autonomy but not social support is associated with IYCF indicators in Northern Ghana. Both maternal social support and autonomy are not associated with child growth indicators. Child survival programmes should incorporate or strengthen women empowerment interventions to improve child nutrition in Northern Ghana.

## Supplementary Information

**Additional file 1: Supplementary Table 1.** Other determinants of infant and young child feeding indicators.

## Data Availability

The datasets used and/or analyzed during the current study are available from the corresponding author on reasonable request.

## Abbreviations

CWC: Child Welfare Clinic
CHPS: Community Health Planning and Services
MAD: Minimum Acceptable Diet
MDD: Minimum Dietary Diversity
MMF: Minimum Meal Frequency
IYCF: Infant and Young Child Feeding
