# Supplementary material for: Maternal autonomy but not social support is a predictor of child feeding indicators in the Northern Region, Ghana
Source: BMC Nutr. 2022 Nov 18;8:135. doi: 10.1186/s40795-022-00630-8 (PMC9673434; doi:10.1186/s40795-022-00630-8)
Supplement: Supplementary file 1 — Additional file 1: Supplementary Table 1. Other determinants of infant and young child feeding indicators. [file 40795_2022_630_MOESM1_ESM.docx]

**Supplementary Table 1: Other determinants of infant and young child feeding indicators**

| **Variable** |  | **Minimum dietary diversity** | | **Minimum meal frequency** | | **Minimum acceptable diet** | |
| --- | --- | --- | --- | --- | --- | --- | --- |
|  | **Total** | **Yes (%)** | **P-value** | **Yes (%)** | **P-value** | **Yes (%)** | **P-value** |
| **Low birth weight** |  |  | 0.080 |  | 0.094 |  | 0.848 |
| Yes | 88 | 61.4 |  | 65.9 |  | 45.5 |  |
| No | 307 | 50.8 |  | 74.9 |  | 44.3 |  |
| **Age group of children (months)** |  |  | 0.001 |  | 0.030 |  | <0.001 |
| 6-11 | 258 | 45.0 |  | 68.6 |  | 36.8 |  |
| 12-17 | 79 | 63.3 |  | 81.0 |  | 57.0 |  |
| 18-23 | 58 | 75.9 |  | 81.0 |  | 62.1 |  |
| **Sex of children** |  |  | 0.330 |  | 0.345 |  | 0.419 |
| Male | 184 | 50.5 |  | 70.7 |  | 42.4 |  |
| Female | 211 | 55.5 |  | 74.9 |  | 46.4 |  |
| **Maternal age (years)** |  |  | 0.381 |  | 0.918 |  | 0.212 |
| 15-24 | 132 | 52.3 |  | 72.7 |  | 40.2 |  |
| 25-29 | 139 | 49.6 |  | 71.9 |  | 43.2 |  |
| 30+ | 124 | 58.1 |  | 74.2 |  | 50.8 |  |
| **Marital status** |  |  | 0.456 |  | 0.314 |  | 0.723 |
| In a union | 373 | 53.6 |  | 73.5 |  | 44.8 |  |
| Not in a union | 22 | 45.5 |  | 63.6 |  | 40.9 |  |
| **Educational status** |  |  | 0.830 |  | 0.448 |  | 0.998 |
| No education | 134 | 53.7 |  | 76.9 |  | 44.8 |  |
| Low education | 216 | 53.7 |  | 70.8 |  | 44.4 |  |
| High education | 45 | 48.9 |  | 71.1 |  | 44.4 |  |
| **Religion** |  |  | 0.740 |  | 0.750 |  | 0.504 |
| Christianity | 34 | 55.9 |  | 70.6 |  | 50.0 |  |
| Islam | 361 | 52.9 |  | 73.1 |  | 44.0 |  |
| **Ethnicity** |  |  | 0.158 |  | 0.422 |  | 0.095 |
| Dagomba | 326 | 51.5 |  | 72.1 |  | 42.6 |  |
| Others | 69 | 60.9 |  | 76.8 |  | 53.6 |  |
| **Type of building** |  |  | 0.591 |  | 0.053 |  | 0.975 |
| Block | 310 | 53.9 |  | 70.6 |  | 44.5 |  |
| Others | 85 | 50.6 |  | 81.2 |  | 44.7 |  |
| **Household wealth index (tertile)** |  |  | 0.161 |  | 0.233 |  | 0.158 |
| Poorest | 133 | 48.9 |  | 67.7 |  | 38.3 |  |
| Medium | 132 | 59.8 |  | 76.5 |  | 50.0 |  |
| Richest | 130 | 50.8 |  | 74.6 |  | 45.4 |  |
